# Mood disorder as a manifestation of primary hypoparathyroidism: a case report

**DOI:** 10.1186/1752-1947-8-326

**Published:** 2014-10-03

**Authors:** Regis G Rosa, Alcina JS Barros, Antonio RB de Lima, William Lorenzi, Rafael R Da Rosa, Karine D Zambonato, Gustavo V Alves

**Affiliations:** 1 Internal Medicine Division, Hospital Municipal Getúlio Vargas, Pinheiro Machado 331, Sapucaia do Sul, RS 93210-180, Brazil

**Keywords:** Calcium homeostasis, Depression, Hypoparathyroidism, Mental disorders, Mood disorder

## Abstract

**Introduction:**

Primary hypoparathyroidism is a rare condition caused by parathyroid hormone deficiency and characterized by hypocalcemia. The clinical manifestations of primary hypoparathyroidism include tetany, seizures, paresthesias, dementia, and parkinsonism. Psychiatric manifestations such as mood disorders are unusual and may constitute a major diagnostic challenge, especially if the typical manifestations caused by hypocalcemia are absent.

**Case presentation:**

The patient was a 22-year-old Caucasian man with a history of chronic omeprazole use and periodic seizures, who presented to the emergency department of a secondary hospital in Southern Brazil with symptoms of major depression (sadness, anhedonia, loss of appetite, insomnia, and fatigue) associated with paresthesias affecting his toes. The initial electrocardiogram revealed a prolonged QTc interval. A computed tomography scan of his brain revealed bilateral, nonenhancing hyperdense calcifications involving the putamen and caudate nucleus. An electroencephalogram showed generalized bursts of slow spikes. Blood laboratory study results indicated serum hypocalcemia, hypomagnesemia, and hyperphosphatemia associated with a low parathyroid hormone level. His serum levels of albumin, 25-hydroxyvitamin D, thyroid-stimulating hormone, T3 and T4 thyroid hormones, as well as the results of kidney function tests, were normal. The definitive diagnosis was primary hypoparathyroidism with psychiatric manifestations due to chronic hypomagnesemia induced by proton pump inhibitor use.

**Conclusions:**

In some cases, to differentiate between a primary psychiatric disorder and primary hypoparathyroidism with neuropsychiatric symptoms may represent a challenge given that the classical manifestations of hypocalcemia, especially tetany, may be absent in the setting of chronic hypoparathyroidism. Clinicians and psychiatrists should consider primary hypoparathyroidism part of the differential diagnosis during the evaluation of patients with mood symptoms, especially in the context of atypical presentations associated with hypocalcemia.

## Introduction

Parathyroid hormone (PTH) plays an important role in the metabolism of calcium and phosphate. In response to a decrease in the serum calcium level, parathyroid cells increase PTH secretion physiologically. Through direct action on the kidney and bone cells, and indirect effects on the bowels, PTH increases serum calcium levels and decreases serum phosphate levels. In addition, PTH promotes bone resorption, decreases urinary calcium excretion, enhances the conversion of 25-hydroxyvitamin D to 1,25-dihydroxyvitamin D, and increases intestinal calcium absorption, and renal phosphate excretion [1-3].

Primary hypoparathyroidism is characterized by the absence or decreased secretion of PTH. As a consequence, patients with primary hypoparathyroidism often present clinical manifestations related to hypocalcemia such as tetany, seizures, muscle cramps, paresthesias (affecting the fingertips, toes, and perioral area), and carpopedal spasms. In cases of chronic hypocalcemia, symptoms become less specific, ranging from asymptomatic states to atypical clinical presentations, such as neuropsychiatric manifestations. In this sense, mood disorders, such as depression have been reported rarely as the main manifestation of hypoparathyroidism. The typical laboratory findings of primary hypoparathyroidism include hypocalcemia and hyperphosphatemia in the presence of undetectable or abnormally low levels of PTH. Skull radiographs or computed tomography scans of the brain may reveal symmetrical bilateral basal ganglia calcification. The typical electroencephalogram pattern includes bursts of high-voltage paroxysmal slow waves. Electrocardiography may reveal a prolonged QTc interval and T wave abnormalities owing to hypocalcemia [4-6].

The etiology of primary hypoparathyroidism includes surgical destruction of parathyroid glands (the most common cause), neck irradiation, autoimmune hypoparathyroidism (isolated or combined with other endocrine deficiencies, for example, polyglandular autoimmunity), idiopathic hypoparathyroidism, hypomagnesemia, Wilson’s disease, hemochromatosis, and congenital syndromes (for example, DiGeorge syndrome) [7,8].

Here we describe an unusual case of primary hypoparathyroidism with an atypical predominant manifestation of major depression secondary to hypomagnesemia induced by chronic proton pump inhibitor use. Following the case presentation, we provide a brief discussion and review of previously published data regarding psychiatric manifestations of primary hypoparathyroidism.

## Case presentation

**Figure 1:**
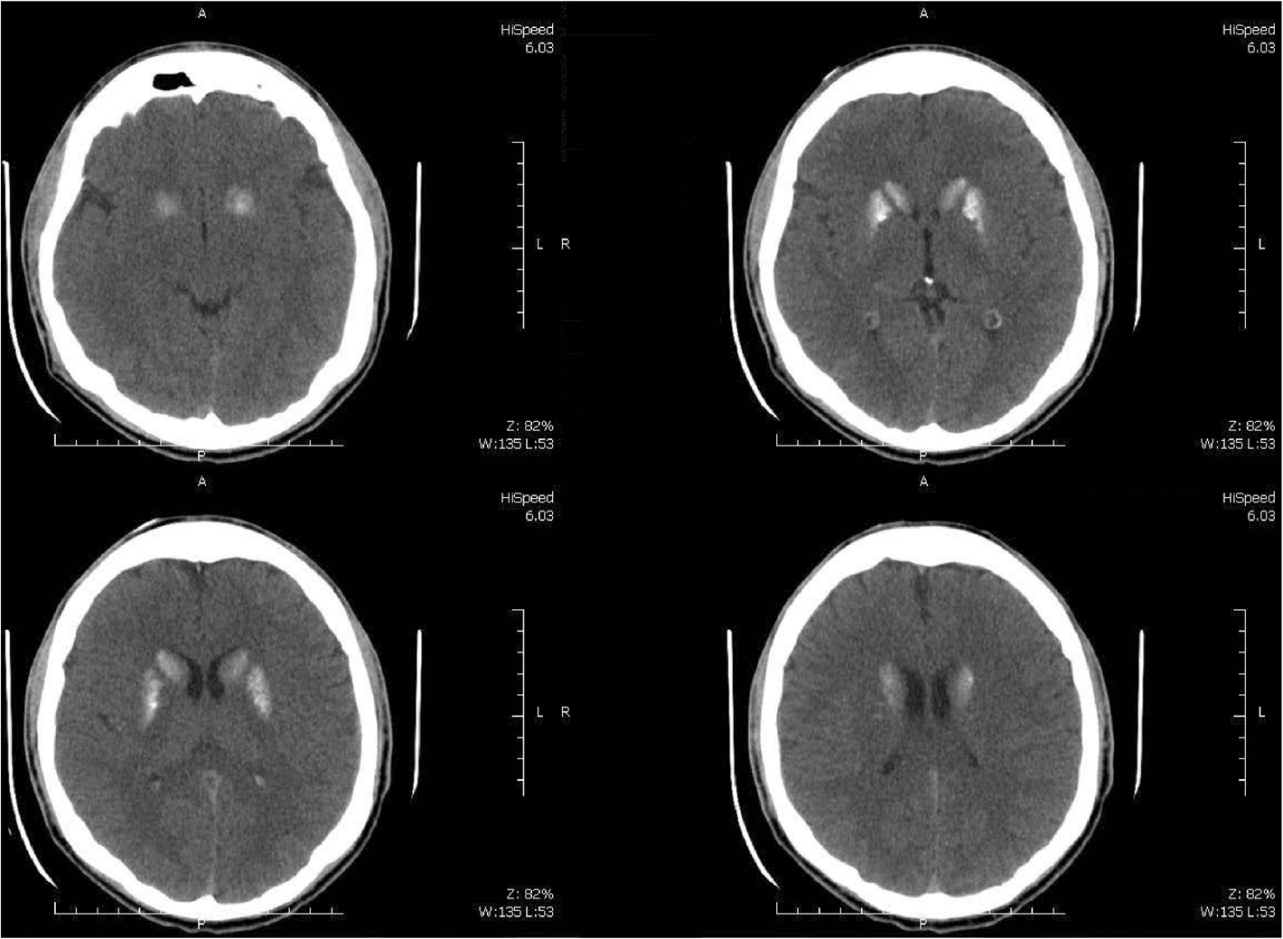
Computed tomography scan of the brain.

**Figure 2:**
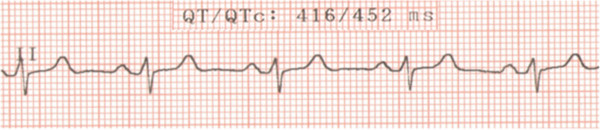
**Electrocardiogram.** Note: Normal QTc interval in men is <430ms.

A 22-year-old Caucasian man with a history of periodic generalized tonic–clonic seizures was admitted to a secondary hospital because of symptoms of major depression. He experienced depressed mood, decreased interest in most activities, loss of appetite (with 10% weight change), insomnia, fatigue, loss of concentration, and thoughts of death (without suicidal ideation) in the previous 3 months. In addition, he complained of paresthesias involving his toes. He had no history of substance abuse and presented no symptoms of mania. He had been undergoing treatment with valproic acid 600mg/day orally for the last 2 years (for seizures treatment) and omeprazole 40mg/day orally during the last 5 years (for dyspepsia treatment). His physical examination was normal. No sensory or motor deficit was found objectively, and Chvostek and Trousseau signs were absent. The psychiatric evaluation was compatible with major depressive symptoms according to the criteria of the *Diagnostic and Statistical Manual of Mental Disorders*, fourth edition, text revision (DSM-IV-TR) [9]. A routine computed tomography scan of his brain revealed bilateral nonenhancing hyperdense calcifications involving the putamen and caudate nucleus in a symmetrical pattern (Figure 1). The electroencephalogram showed bursts of high-voltage paroxysmal slow waves. The electrocardiography demonstrated a prolonged QTc interval without other abnormalities (Figure 2). He had a low total calcium level (5.4mg/dL; normal range 8.8mg/dL to 11.0mg/dL), low ionic calcium level (0.73mg/dL; normal range 1.0mg/dL to 1.3mg/dL), elevated serum phosphate level (6.3mg/dL; normal range 2.5mg/dL to 4.8mg/dL), low intact PTH level (8.3pg/mL; normal range 12.0pg/mL to 88.0pg/mL), and low magnesium level (0.7mg/dL; normal range 1.9mg/dL to 2.5mg/dL). His serum levels of albumin, 25-hydroxyvitamin D, thyroid-stimulating hormone, T3 and T4 thyroid hormones, creatinine, and blood urea nitrogen were normal. His 24-hour urine excretion of magnesium was low (62mg; normal range 75mg to 150mg). His fractional magnesium clearance was 0.8%, suggesting that his renal retention of magnesium was appropriate. A definitive diagnosis was made of primary hypoparathyroidism due to hypomagnesemia with major depressive symptoms as the predominant clinical manifestation. The probable etiology of the hypomagnesemia was the chronic pump proton inhibitor use, given that other causes of chronic hypomagnesemia were absent (that is, malabsorption, alcoholism, diuretic therapy, and tubulointerstitial kidney diseases). Omeprazole was discontinued promptly given that the upper gastrointestinal endoscopy was normal. He received oral treatment with calcium carbonate 3g/day, calcitriol 0.50mcg/day, magnesium pidolate 366mg/day, and sertraline 50mg/day. Of interest, during his hospitalization period of 41 days, his symptoms of major depression began to improve only after correction of total serum calcium levels. He was discharged with normal total calcium (9.5mg/dL) and magnesium levels (2.0mg/dL). His clinical and laboratory improvement was sustained as verified by a routine out-patient medical visit 2 months after hospital discharge.

## Discussion

The present report describes a case of primary hypoparathyroidism as the unusual etiology of major depressive symptoms. The chronic course of the disease probably contributed to its atypical presentation, which consisted of depressive mood symptoms predominantly. Because of the high prevalence of depressive syndromes, this case alerts us to the need of investigating clinical causes intensively before diagnosing a patient with a primary mental disorder. Furthermore, a delay in the identification of the underlying cause could represent serious consequences for the patient, culminating in behavioral manifestations and increased risk of death.

The DSM-IV-TR [9] criteria state that a mood disorder can be a result of a medical condition, exemplified by Parkinson’s disease and hypothyroidism. The DSM-IV-TR also states that “the criteria for the diagnosis are similar to those for major depressive episode or a manic episode; however, the full criteria for these diagnoses need not be met. It is important to establish if the depressive symptoms are a direct physiological result of the medical condition, instead of a psychological response to a medical problem”.

The relationship between hypoparathyroidism and psychiatric syndromes is supported by previous studies. A study by Aggarwal *et al*. [10] demonstrated an association between idiopathic hypoparathyroidism and neuropsychological dysfunction, which was correlated with the duration of illness, female sex, serum calcium, and calcium-phosphorus product during follow-up but not with intracranial calcifications. These results alert us to the fact that patients with hypoparathyroidism may have psychiatric manifestations even in the absence of brain calcifications. Moreover, distinct psychiatric manifestations, such as depression, psychosis, and anxiety in the context of hypoparathyroidism, have also been reported [11-16] (Table 1). Among all of these reported cases, psychiatric manifestations seemed predominant in settings of long-term hypoparathyroidism. Of interest, a clinical history of seizures was common among most reported cases, which may represent an important clue to the suspicion of hypocalcemia and hypoparathyroidism. In addition, psychiatric symptoms were characteristically resistant to conventional psychopharmacological treatment (for example, antidepressants and antipsychotics) until the serum calcium levels were corrected. These findings underscore the importance of establishing the primary cause of major depressive symptoms, given that the proper treatment of the baseline medical condition may be essential for the control of the psychiatric symptoms.

**Table 1:** Published cases of primary hypoparathyroidism with predominant psychiatric manifestations

**Author [reference], year**	**Patient age, years**	**Sex**	**Psychiatric manifestation**	**Etiology of hypoparathyroidism**
Ang *et al*. [11], 1995	15	Female	Psychosis	Idiopathic
Ilievski *et al*. [12], 2002	26	Female	Paranoid schizophrenia	Idiopathic
Bohrer and Krannich [13], 2007	68	Male	Major depression	Surgical destruction of parathyroids
Patil *et al*. [14], 2010	30	Female	Psychosis	Idiopathic
Bertola *et al*. [15], 2013	51	Female	Chronic psychosis	DiGeorge syndrome
Mirhosseini *et al*. [16], 2013	28	Female	Panic attacks	Surgical destruction of parathyroids
Present study	22	Male	Major depression	Hypomagnesemia

## Conclusions

Patients with chronic hypocalcemia secondary to primary hypoparathyroidism may present clinically with predominant psychiatric symptoms. Primary hypoparathyroidism should be considered part of the differential diagnosis of patients with psychiatric syndromes such as depression, anxiety, or psychosis, especially when the clinical presentation is atypical and associated with abnormalities of calcium metabolism. Routine serum calcium measurement may represent an important tool for the identification of such cases.

## Consent

Written informed consent was obtained from the patient for publication of this case report and any accompanying images. A copy of the written consent is available for review by the Editor-in-Chief of this journal.

## Abbreviations

DSM-IV-TR: *Diagnostic and Statistical Manual of Mental Disorders*, fourth edition, text revision; PTH: Parathyroid hormone.

## Competing interests

The authors have no financial disclosures or conflicts of interest to declare.

## Authors’ contributions

RGR, AJSB, ARBL, WL, RRR, KDZ and GVA arrived at the diagnosis of the patient and management, literature review, and writing of the final manuscript. All authors read and approved the final manuscript.

## Authors’ information

RGR and GVA are internal medicine physicians; AJSB is a psychiatrist; ARBL and WL are surgeons; RRR and KDZ are emergency physicians.
